# Synthesis and Structural Characterization of Selenium Nanoparticles–*Bacillus* sp. MKUST-01 Exopolysaccharide (SeNPs–EPS) Conjugate for Biomedical Applications

**DOI:** 10.3390/biomedicines11092520

**Published:** 2023-09-12

**Authors:** Thirumalaivasan Ramachandran, Devaprakash Manoharan, Sivakumar Natesan, Shyam Kumar Rajaram, Ponmurugan Karuppiah, Mohammed Rafi Shaik, Mujeeb Khan, Baji Shaik

**Affiliations:** 1 Department of Molecular Microbiology, School of Biotechnology, Madurai Kamaraj University, Madurai 625021, Tamil Nadu, India; thivasan7498@gmail.com (T.R.); devprakashm3@gmail.com (D.M.); 2 Department of Biotechnology, Kamaraj College of Engineering and Technology, Virudhunagar 625701, Tamil Nadu, India; kingshyam2003@gmail.com; 3 Department of Botany and Microbiology, College of Science, King Saud University, P.O. Box 2455, Riyadh 11451, Saudi Arabia; pkaruppiah@ksu.edu.sa; 4 Department of Chemistry, College of Science, King Saud University, P.O. Box 2455, Riyadh 11451, Saudi Arabia; kmujeeb@ksu.edu.sa; 5 School of Chemical Engineering, Yeungnam University, Gyeongsan 38541, Republic of Korea; shaikbaji@yu.ac.kr

**Keywords:** exopolysaccharide, selenium nanoparticles, SeNPs–EPS conjugate, antioxidant activity, gnotobiotic assay

## Abstract

Exopolysaccharides (EPS) are exogenous microbial metabolites generated predominantly during the development of bacteria. They have several biological potentials, including antibacterial, antioxidant, and anticancer actions. Polysaccharide-coated nanoparticles have high biological activity and are used in treatments and diagnostics. In this research, selenium nanoparticles (SeNPs) are synthesized and conjugated with bacterial (*Bacillus* sp. MKUST-01) exopolysaccharide (EPS). Initially, the creation of SeNPs conjugates was verified through UV–Vis spectral examination, which exhibited a prominent peak at 264 nm. Additionally, X-ray diffraction (XRD) analysis further substantiated the existence of crystalline Se, as evidenced by a robust reflection at 29.78°. Another reflection observed at 23.76° indicated the presence of carbon originating from the EPS. Fourier transform infrared spectroscopy (FT-IR) analysis of the EPS capped with SeNPs displayed characteristic peaks at 3425 cm^−1^, 2926 cm^−1^, 1639 cm^−1^, and 1411 cm^−1^, corresponding to the presence of O-H, C-H, C=O, and COO–groups. The SeNPs themselves were found to possess elongated rod-shaped structures with lengths ranging from 250 to 550 nm and a diameter of less than 70 nm, as confirmed using scanning electron microscopy and particle size analysis. In contrast to the SeNPs, the SeNPs–EPS conjugates showed no hemolytic activity. The overall antioxidant activity of SeNPs–EPS conjugates outperformed 20% higher than SeNPs and EPS. Additionally, experimental observations involving gnotobiotic *Artemia nauplii* experiments were also recorded, such as the supplementation of EPS and SeNPs–EPS conjugates corresponding to enhanced growth and increased survival rates compared to *Artemia nauplii* fed with SeNPs and a microalgal diet.

## 1. Introduction

The biological synthesis of nanoparticles has garnered significant attention over the last few decades due to their unique physical stability, biological attributes, and their promising applications in the field of biomedicine [1]. While rapid synthesis methods such as laser ablation, pyrolysis, lithography, chemical vapor deposition, sol–gel, and electrodeposition are efficient, they come with elevated expenses and the possibility of posing health risks to humans [2]. Conversely, the utilization of microorganisms, algae, and plant components for the eco-friendly production of nanoparticles has demonstrated greater efficacy compared to chemical methods [3]. Notably, the biosynthesis of nanoparticles such as copper, silver, gold, palladium, platinum, and zinc oxide has found diverse applications in diagnostics and therapeutics [1]. For instance, nanoparticles encapsulated or adsorbed within matrix materials have facilitated the delivery of therapeutic molecules [4].

Among the array of metallic elements, elemental selenium (Se) holds special significance in scientific exploration [5]. Present in both inorganic (selenite and selenate) and organic (selenomethionine and selenocysteine) forms, selenium plays a crucial role as a micronutrient in various living organisms, including humans [6,7]. Its presence is observed in crystalline and amorphous polymorphic structures, each carrying distinct roles. In the organic form, found in selenoproteins, selenium exhibits essential functions in animals, including enzyme activity, immune response, reproduction, and pro- and anti-oxidative properties [8,9]. Notably, selenium acts as a cofactor within selenoproteins, with the well-known glutathione peroxidase (GSH-Px) being the first identified selenoprotein. GSH-Px catalyzes the conversion of hydroperoxides to alcohols [10]. However, excessive concentrations of selenium can exert toxicity on cellular metabolism [11]. Nano-selenium, as opposed to metal selenium, displays reduced toxicity and heightened enzyme activity (e.g., GSH-Px and thioredoxin reductase), enhancing immune functions [12,13].

Polysaccharides have emerged as promising candidates for the creation of biologically inspired nanocomposites [14,15]. Exopolysaccharides (EPS) are exogenous metabolites synthesized during bacterial, microalgal, plant, and animal growth and exhibit remarkable potential in pharmaceuticals and drug development. Through novel manufacturing or modification techniques, the properties of polysaccharides can be enhanced, expanding their application in food and medicine [16,17]. With a diverse range of chemical structures, EPS is believed to provide self-protection against antimicrobial agents. EPS derived from beneficial gut bacteria can modulate the host’s gut microbiota, thereby promoting intestinal health, immune modulation, and improved gut function [18,19]. Probiotic bacteria like *Bacillus*, *Lactobacillus*, *Bifidobacterium*, and *Lactococcus* produce EPS, which comes in either homopolysaccharide or heteropolysaccharide forms, each offering distinct biological functions [20]. Despite this, the synthesis of nanoparticles using bacterial EPS and their biomedical applications remain underexplored.

Typically, chemical reduction involving a reducing agent and stabilizer constitutes the primary synthetic approach for producing SeNPs. However, the use of chemical stabilizers can hinder their applicability in biological contexts due to their inherent toxicity [21]. This study aims to investigate how bacterial EPS conjugation influences the structural attributes and biological properties of SeNPs. Employing a reductive oxidation approach with ascorbic acid, SeNPs were synthesized, stabilized, and subsequently conjugated with bacterial EPS extracted from *Bacillus* sp. MKUST-03. Functional properties of both SeNPs and EPS-conjugated SeNPs were assessed through antibacterial, antioxidant, anti-inflammatory, and cell toxicity assays, building upon previous research findings.

## 2. Materials and Methods

### 2.1. Isolation and Screening of EPS-Producing Bacteria

The silt samples were collected from four different locations such as Manakudi estuary, Kanyakumari (8°088′ N, 77°486′ E), Mandabam, Ramanathapuram (9.25° N 79.3° E), Vaigai River, Madurai (9°205′ N, 79°000’ E), and Checkanurani lake, Madurai (10.023′ N, 78.223′ E), Tamil Nadu, South India, were brought to the laboratory for microbiological analysis. A standard serial dilution method was completed with sterile saline blanks and subsequently 0.1 mL these of diluted sample was spread on nutrient agar medium supplement with 3% sucrose and then plates were incubated at 37 °C for 48 h. Distinct colonies with varying morphologies, i.e., different mucopurulent colonies, were selected and sub-cultured on nutrient agar medium containing 0.08% Congo red dye and 3% sucrose. The plates were incubated for 48 h at 37 °C. The black color colony on Congo red plates confirmed the production of EPS [22]. Highly mucoidal isolates were further cultured in nutrient broth with 2% sucrose for 48 h at 37 °C to select high EPS-producing isolate.

### 2.2. Identification of EPS-Producing Bacteria

Bacterial strain EPSB-03, assigned as MKUST-01, underwent a comprehensive screening and characterization process such as morphological and physiological assessments based on Bergey’s manual of determinative bacteriology, as well as molecular analysis via 16S rRNA gene sequencing methods [23]. For molecular analysis, the strain MKUST-01 DNA was extracted using the phenol–chloroform method and the 16S rRNA gene was amplified with the following common primers: FP, 5′–AGAGTTTGATC CTGGCTCAG–3′, and RP, 5′(CGTTACCTTGTTACGACTT–3′. Following PCR, the resulting products were purified and subjected to sequencing by Biokart India Pvt Ltd., Bengaluru, India, Sequence homology was evaluated through GenBank, employing the CLUSTAL X software V 2.0 (NCBI). Finally, the phylogenetic tree was constructed by neighbor-joining method by using MEGA 6.0 software [24].

### 2.3. Production, Extraction, and Purification of EPS

The OD value of 0.1 at 600 nm of strain MKUST-01 was inoculated into a 250 mL conical flask containing 100 mL of nutrient broth. It was then incubated in a temperature-controlled orbiter shaking incubator at 100 rpm for 48 h at 37 °C. Afterward, the cell-free culture supernatant was obtained by centrifugation at 8000 rpm for 15 min. Cold absolute alcohol was added to the supernatant in a 1:2 (*v*/*v*) ratio, and the mixture was left at 4 °C overnight to facilitate the precipitation of EPS. The resulting precipitate was re-precipitated using a 1:2 volume of cold absolute alcohol, following resuspension in Milli-Q water. This process was repeated twice, and the precipitate was then dried at 50 °C. The total EPS content was quantified using phenol–sulfuric acid method, with glucose serving as the standard [25]. Further, partial purification of the EPS was accomplished using the TCA precipitation method reported by Yang et al. [26]. Subsequently, the purified sample was lyophilized and utilized for physicochemical analysis.

### 2.4. Synthesis of Selenium Nanoparticles (SeNPs)

SeNPs were synthesized through the reduction of sodium selenite by ascorbic acid and were subsequently stabilized using Tween-20, following a procedure adapted from Vahdati and Tohidi Moghadam [27]. Briefly, 30 mg of Na_2_SeO_3_·5H_2_O was dissolved in 90 mL of Milli-Q water. Ascorbic acid (10 mL, 56.7 mM) was slowly added drop by drop to the sodium selenite solution with vigorous stirring, and 10 µL of polysorbate added after every 2 mL of ascorbic acid. This process led to the formation of SeNPs, visually indicated by a noticeable shift in color to a clear red. All solutions were prepared in a sterile environment with double-distilled water. Subsequently, the solution was subjected to centrifuged at 12,000 rpm to isolate the SeNPs, which were then dried at 50 °C.

### 2.5. Exopolysaccharide–Nanoparticle Conjugation

A 100 mg quantity of EPS was dissolved in 5 mL of Milli-Q water with constant 600 rpm stirring for 30 min at room temperature for 30 min. Then, 5 mL colloidal SeNPs (20 mg/mL) solution was slowly added into EPS solution and vigorously vortexed for 10 min, followed by sonication for 30 min in a bath (60 °C) Sonicator (SOLTEC Sonica, Ultrasonic Cleaners Systems, Milano, Italy) [28].

### 2.6. Characterization of SeNPs–EPS

Characterization of partially purified EPS, SeNPs, and SeNPs–EPS was determined by different spectroscopic analyses. All the samples were characterized by UV–Vis absorption spectrophotometer (Systronics UV-VIS spectrophotometer 117, Ahmedabad, Gujarat, India), and spectra were recorded within 200–600 nm wavelength. The functional groups were identified using infrared spectroscopy (FTIR; Perkin Elmer 1,000 FT-IR spectrometer, Waltham, MA, USA). Concisely, a mixture containing EPS, SeNPs, SeNPs–EPS, and KBr in a 1:100 ratio was compressed into a disc using a hydraulic press. This disc was then subjected to monitoring across the wavelength ranging from 4000 to 400 cm^−1^. The structural properties of the isolated EPS were determined using nuclear magnetic resonance (NMR) spectroscopy, utilizing deionized water (D_2_O) containing 0.1% tetramethylsilane as an internal standard. The ^1^H and ^13^C NMR spectra of EPS were acquired using an NMR spectrometer (Bruker Advance III HD Nanobay 400 MHz FT-NMR Spectrometer, Allentown, PA, USA) equipped with a cryogenically cooled ^1^H and ^13^C NMR detection probe (Bruker Topspin, Allentown, PA, USA). Chemical shifts were referenced to D_2_O (δH 4.65), expressed in parts per million (ppm), and coupling constants were measured in Hertz.

TGA analysis of EPS was conducted using a thermal analyzer, Trios V5.2.2.47561, New Castle, DE, USA. In summary, the dried samples were placed into an Al_2_O_3_ crucible and subjected to a linear heating rate of 10 °C per min, ranging from 10 °C to 800 °C. These experiments were carried out in an air atmosphere with a flow rate of 100 mL per min. For DSC analysis, 2.0 mg of the sample was sealed in an aluminum pan, and the melting point and enthalpy change were determined, with an empty pan serving as a reference. The heating rate employed was 10 °C/min, spanning from 10 to 800 °C [29]. The surface morphology and microstructure of EPS, SeNPs, and SeNPs–EPS were examined using scanning electron microscopy (SEM), specifically a Quanta FEG 250, Waltham, MA, USA. Accordingly, 5 mg of samples was applied to a carbon-coated stub, sputtered with gold, and images were acquired using VEGA 3.0 TE Scan SEM (Waltham, MA, USA) at an accelerating voltage of 10 kV. The physical characteristic of samples were assessed using X-ray diffraction (XRD) analysis, utilizing a powder diffractometer.

### 2.7. Biological Activities

#### 2.7.1. Reducing and Scavenging Activity

The ferric-reducing assay was carried out following the method outlined by Akgul et al. [30]. Additionally, the DPPH free-radical scavenging activity was assessed using the procedure described by Shimada et al. [31]. To elaborate, a 0.1 mM of DPPH solution in 100% methanol (prepared freshly) was utilized. A 1 mL volume of this solution was mixed with 4 mL of the sample in 40% methanol, encompassing various concentrations (0.2, 0.4, 0.6, 0.8, and 1.0 mg/mL of EPS) and allowed to react in darkness for 30 min. The activity was measured at 517 nm against blank using a spectrophotometer (Shimadzu, Kyoto, Japan). Lower absorbance in the reaction mixture indicated higher free-radical scavenging activity and l-ascorbic acid serving as a standards. The % of free radical scavenging activity was calculated using the following formula:

% Scavenging activity = [(control OD − sample OD)/control OD] × 100(1)

#### 2.7.2. Hemolytic Activity

To assess hemolytic activity, the method outlined by Younis et al. [32] was employed. A blood sample was obtained from a healthy volunteer using heparinized tubes and subsequently washed twice with phosphate-buffered saline (1× PBS, pH 7.4). In a sterile tube, 1 mL of EPS solutions at varying concentrations (25, 50, 100, 200, and 500 g/mL) was mixed with 1 mL of 10% red blood cell (RBC) suspension. After 1 h of incubation at room temperature, the cell suspensions were subjected to centrifugation for 10 min at 1500 g. Following centrifugation, the supernatants were transferred to flat-bottom 96-well plates, and their absorbance (A) was measured at 492 nm using an ELISA reader (BioRad, Hercules, CA, USA). Later, the % of hemolytic activity was calculated with following formula:

(2) $$\mathrm{Hemolytic}\;\mathrm{activity}\;\left(\%\right)=\frac{\mathrm{Absorption}\;\mathrm{by}\;\mathrm{sample}\;-\;\mathrm{Absorption}\;\mathrm{by}\;\mathrm{negative}\;\mathrm{control}}{\mathrm{Absorption}\;\mathrm{by}\;\mathrm{positive}\;\mathrm{control}\;-\;\mathrm{Absorption}\;\mathrm{by}\;\mathrm{negative}\;\mathrm{control}}\times 100$$

#### 2.7.3. Cell Viability using MTT Assay

Human embryonic kidney cells (HEK-293) were procured from the American Type Culture Collection Centre (ATCC, Manassas, VA, USA) in 10% fetal bovine serum, 50 units/mL of penicillin/streptomycin supplemented Dulbecco Modified Eagle’s Medium (DMEM, HiMedia). Cytotoxicity assessments of EPS, SeNPs, and SeNPs–EPS were conducted using the 3-(4,5-dimethylthiazol-2-yl)-2,5-diphenyltetrazolium bromide (MTT) reduction assay [33]. In brief, HEK-293 (1.5 × 10^4^ cells/well) were seeded into 96-well plates, and incubated for 24 h at 37 °C in an environment with 5% CO_2_ and 95% relative humidity. Different concentrations of EPS, SeNPs, and SeNPs–EPS ranging from 10 to 200 µg/mL in 200 μL volumes were added to the basal DMEM in each well, followed by re-incubation. Cells treated with 0.25% dimethyl sulfoxide (DMSO) served as a negative control. After 24 h of incubation, 100 μL of freshly prepared MTT solution (1 mg/mL) was added to each well and further incubated for 4 h. Finally, 100 μL of DMSO was added and incubated for 30 min at room temperature. The absorbance of the formazan product was measured at 595 nm using a microplate reader (Imark, Bowie, MD, USA; Biorad, Hercules, CA, USA). The percentage of cell viability was determined using the following formula:

Cell viability (%) = [(test/control) × 100)](3)

#### 2.7.4. *In Vivo* Toxicity Assay

Toxicity assessment of EPS, SeNPs and SeNPs–EPS was conducted using *Artemia nauplii* larvae, a commonly employed model organism in the toxicity assessment [34]. *Artemia nauplii* cysts (Horizone Fish Foods, Palakkad, Kerala, India) were used in the experiments. These cysts were hydrated and decapsulated following the method described by Kumar et al. [35] with slight modifications and maintained under aseptic conditions. Concisely, various concentrations (50, 100, and 200 µg/mL) of the test samples were prepared in artificial seawater (35 g/L, NaCl). Subsequently, 100 µL of each tested concentration was added to individual wells of 24-well microtiter plates and then 10 Artemia larvae were introduced into each well. The length of individual larvae was measured on days 1, 2, and 3. Morphometric analysis was carried out with three replicates per treatment using an Olympus Binocular Microscope-CX21 at ×40 magnification, and survival rates were recorded for each replicate by using the formula;

Survival (%) = (number of swimming larvae survived/number of larvae stocked) × 100(4)

### 2.8. Statistical Analysis

The experimental results were presented in the form of mean values along with their corresponding standard deviations (SD). Group comparisons were conducted through a one-way analysis of variance (ANOVA) followed by Tukey’s multiple comparison post hoc test. Statistical analyses were carried out using GraphPad Prism statistical software in Windows platform (San Diego, CA, USA).

## 3. Results and Discussion

### 3.1. Isolation and Identification of EPS-Producing Bacteria

**Figure 1:**
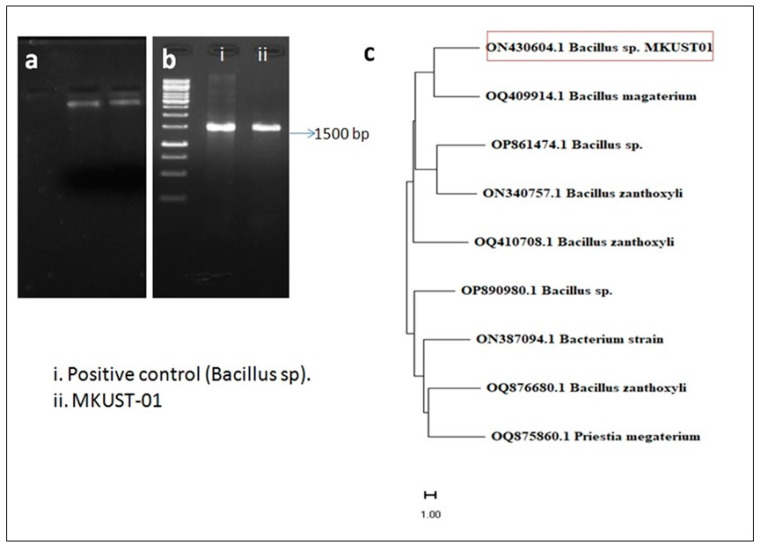
Gel electrophoresis and Phylogenetic tree of *Bacillus* sp. MKUST-01; (**a**) Genomic DNA, (**b**) Marker and PCR product, (**c**) phylogenetic tree of MKUST-01 strain.

A total of thirty (*n* = 30) morphologically distinctive bacterial colonies were isolated from sediment soil samples. The string test and Congo red agar plates showed six isolates produced promising EPS (Supplementary Figure S1). Upon quantitative analysis of EPS from the isolates (Table 1), MKUST01 produced a high amount of EPS (3.37 g/L), and the partially purified EPS showed 89% (*w*/*w*) carbohydrate content, and it was significantly higher than other EPS isolates given in Table 1. The isolate MKUST01 was morphologically and biochemically identified as *Bacillus* sp. and confirmed using 16S rRNA gene sequencing and phylogenetic analysis as shown in Figure 1. The isolate MKUST-01 was identified with 99% sequence similarity with *Bacillus* sp. (GenBank accession number: ON430604). A high EPS-producing bacteria *Bacillus* sp. MKUST-01 was screened from the sediment soil of Manakudi estuary, Kanyakumari District, Tamil Nadu, and used for EPS production.

### 3.2. Characterization of Conjugated SeNPs–EPS

The synthesis of SeNPs was accomplished through the reduction of SeO_3_^2−^ using ascorbic acid, resulting in the formation of elemental selenium (Se^0^). Wet chemical-based nanoparticle syntheses offer favorable reducing properties and biocompatibility for nanomaterials [36]. These are bottom-up approaches in which particles at the atomic or molecular level are integrated to form nanostructures [37]. They are simple, modular, and scalable methods, which are effective in producing controlled nanomaterials and are essential for the optimization of optical, electronic, and surface properties [38]. Therefore, using the wet-chemical approach, in this study, the production of monodisperse SeNPs was achieved by reducing Na_2_SeO_3_ with ascorbic acid as a reducing agent and TritonX 100 as the stabilizing agent facilitating the creation of red-colored nanoparticles with an optical property of 265 nm (λmax). This outcome aligns with the findings of Shubharani et al. [39], on the synthesis of SeNPs using ethanol extracts of propolis.

**Figure 2:**
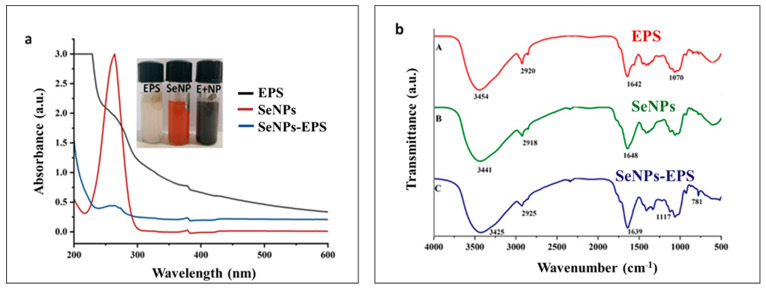
(**a**) Absorption spectrum of EPS, SeNPs, and SeNPs–EPS (inner image showed the colloidal suspension of EPS, SeNPs, and SeNPs–EPS). (**b**) FTIR spectrum of EPS (A), SeNPs (B), and SeNPs–EPS (C).

Figure 2 displays the SeNPs distinctive sharp absorption peak at 268 nm and the intense brick-red colour of the colloidal dispersion. The crystalline nature of SeNPs exhibits absorption maxima at 250 to 280 nm [39,40], and it was similar to our preparations. SeNPs–EPS conjugate shows maximum absorption at 254 nm without a sharp beak (Figure 2), which shows the presence of organic residues (EPS) along with SeNPs. A slight alteration in the λmax (265 nm) of SeNPs–EPS can be attributed to certain unfavorable structural modifications in the biomolecular component during conjugation. Consequently, this may lead to adjustments in the interaction and surface adsorption between the biomolecules and nanoparticles [41,42]. In some cases, the functional properties of nanoparticles have been increased due to altered biophysical and biochemical characteristics [27]. For instance, in the ‘nano–bio’ systems, several dynamic physicochemical interactions, kinetics and thermodynamic exchanges occur at the interface between the surfaces of nanoparticles and biological components like proteins, membranes, etc. [43]. In these scenarios, the surface properties of the nanomaterials that are often govern by chemical composition, functionalization, crystallinity, and morphology of the materials are particularly crucial in determining the resulting interaction of the nano-bio components [44].

Figure 2b represents the FTIR spectra of SeNPs, EPS, and conjugated SeNPs–EPS. The distinctive stretching vibrations associated with the hydroxyl group (-OH) were observed in both spectra. However, the absorption bands of -OH at 3454 nm in Figure 2b (A) shifted slightly to 3425 nm in Figure 2b (C). These findings indicate the presence of some weak interactions between SeNPs and EPS [45]. Furthermore, the appearance of absorption bands in Figure 2b (C) at 2925 nm, 1117 nm, and 781 nm was attributed to the stretching vibrations of aliphatic C-H, C-O-H, and α-D-glucose in the polysaccharide, respectively. Notably, the intensity of the C-O-H band, which is closely associated with short-range molecular interactions in polysaccharides, was reduced and appeared at a higher wave number (approximately 1117 cm^−1^) compared to that of pure EPS (1070 cm^−1^). These observations suggest that some OH groups from EPS were involved in conjugation with Se, disrupting hydrogen bonds within the native EPS and forming new C-O-Se bonds [46]. The presence of various functional groups underscores the role of the reducing agent ascorbic acid and EPS to stabilizing the preparation [47,48].

**Figure 3:**
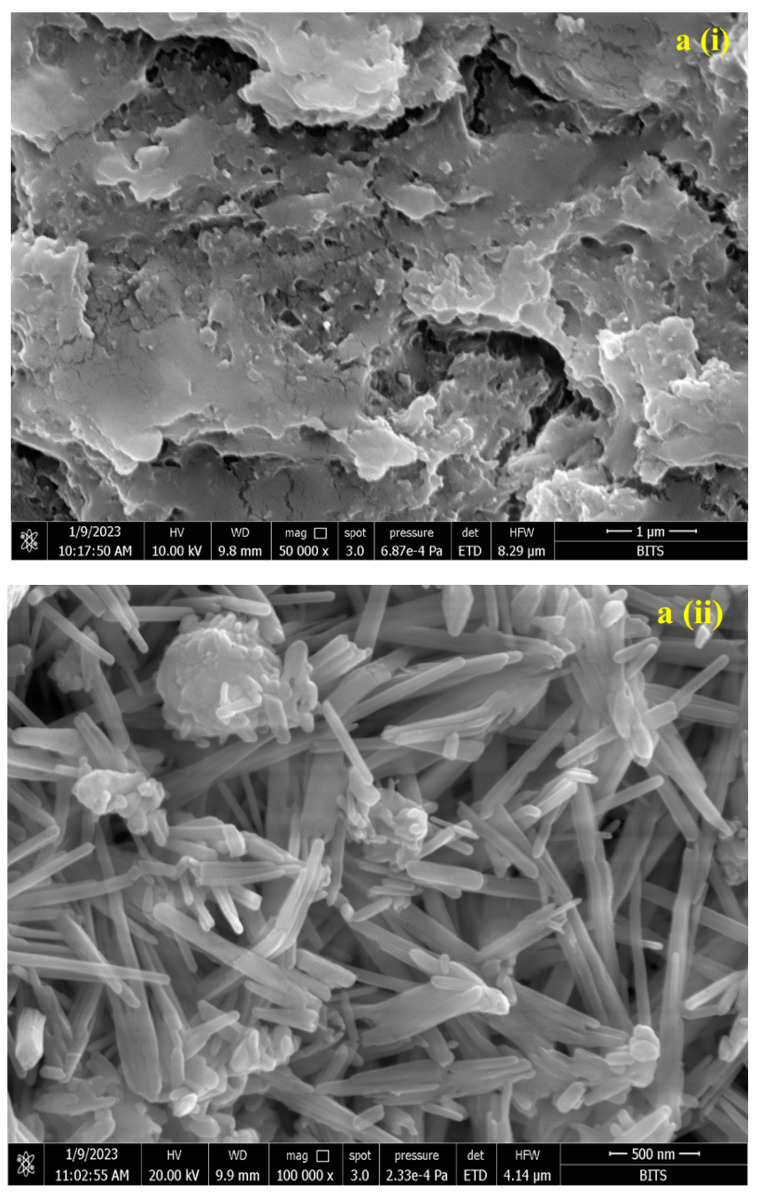

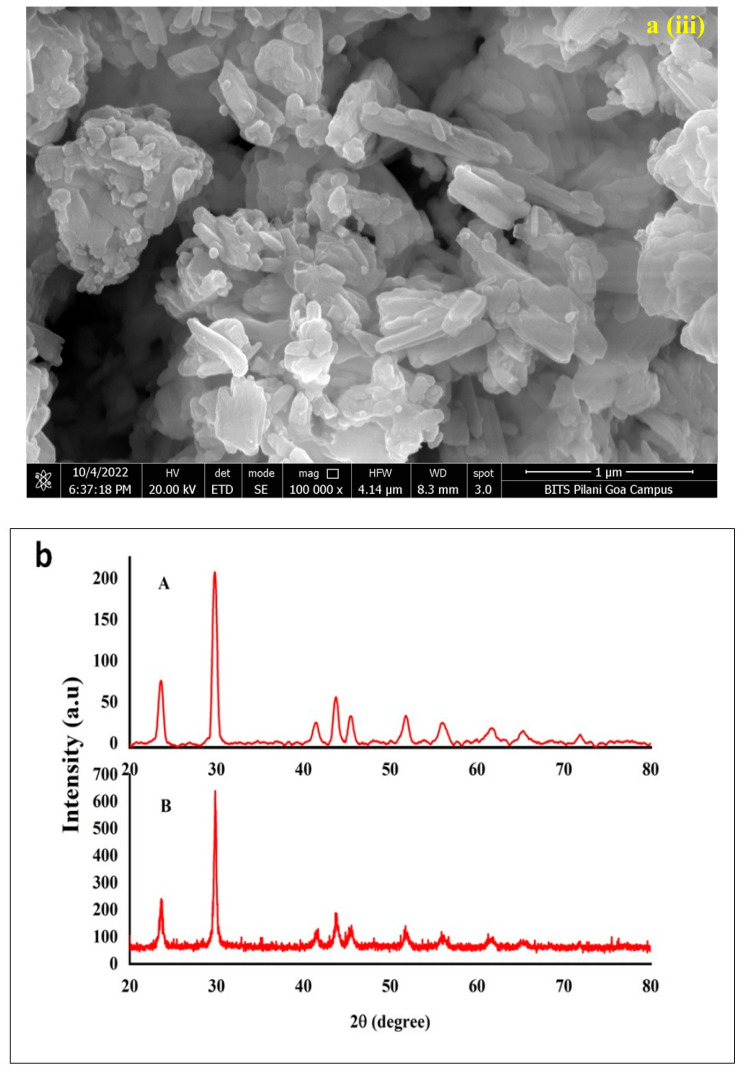
(**a**) SEM image reveals the morphology and size of EPS (**i**), SeNPs (**ii**), and SeNPs–EPS (**iii**). (**b**) X-ray diffraction pattern displays synthesized SeNPs (A) and SeNPs–EPS (B).

SEM images of EPS (Figure 3ai) showed compact, irregular porous, and stacked flakes of polysaccharide of *Bacillus* sp. MKUST-01. This kind of bacterial EPS helps the bacterial cells to adhere to each other due to the high-affinity interactions, which could play a vital role in conjugation with new substances [49,50]. The SeNPs show an amorphous spherical aggregate together with long rod-shaped particles (Figure 3aii), similar to the results of Zhang et al. [51]. These rod-shaped SeNPs are in the size ranging from 250 to 550 nm in length with a diameter of less than 70 nm. On the other hand, the morphology of the SeNPs–EPS showed flake-like morphology (Figure 3aiii), which is possibly due to the aggregation of rod-shaped SeNPs by virtue of the presence of EPS on their surface. The particles of SeNPs–EPS were connected and formed a regular colloidal structure. The earlier study demonstrate that the conjugation of polysaccharide with nanoparticle leads to notable improvements in the physicochemical and functional characteristics of these conjugate complexes [51].

In this study, the XRD pattern suggested that SeNPs is nanocrystalline (Figure 3b) in nature, and it closely resembles the synthesized SeNPs reported in the literature, i.e., as reported during the synthesis of SeNPs with *Diospyros montana* extract [52] and propolis ethanol extracts [39]. For example, the XRD diffractogram of SeNPs and SeNPs–EPS, as depicted in Figure 3b, displays distinct characteristic diffraction peaks at angles of 23.9°, 30.0°, 41.7°, 44.0°, 45.7°, 52.0°, 56.4°, 62.2°, 65.5°, and 68.4°. These peaks correspond to the (100), (101), (110), (102), (111), (201), (003), (202), (210), and (211) reflections of the hexagonal phase of selenium crystals, in accordance with the JCPDS 06-0362 standard [53]. On the other hand, the XRD of SeNPs–EPS conjugates also showed the same peaks, but these peaks are relatively less intense with some background noise, which might be caused by the presence of amorphous exopolysaccharide. Xia et al. proposed that the binding SeNPs with EPS led to the formation of amorphous SeNPs [54].

**Figure 4:**
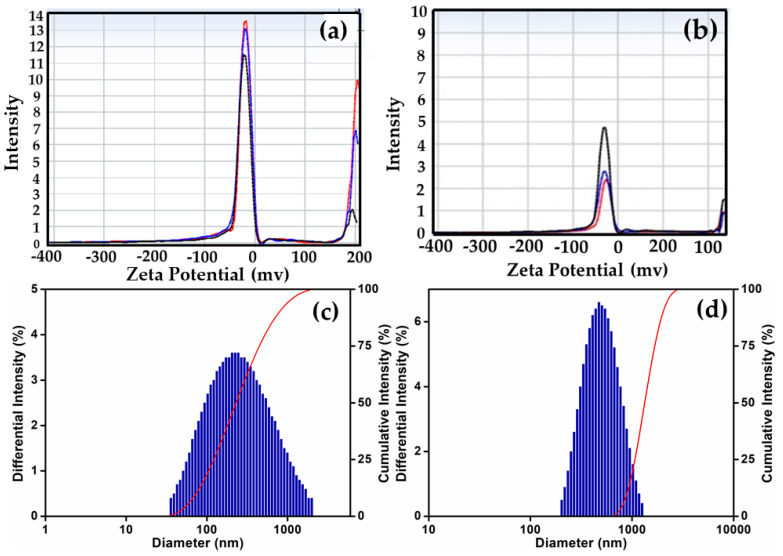
Zeta potential (**a**,**b**) and DLS (**c**,**d**); (**a**) SeNPs zeta potential, (**b**) SeNPs–EPS conjugate zeta potential, (**c**) SeNPs DLS, (**d**) SeNPs-EPS conjugate DLS (Note: Red, blue and block colour peaks are mean average of SeNPs).

The hydrodynamic size of the purified SeNPs and their conjugated state was assessed through the dynamic light scattering (DLS). Figure 4 displays the Z-average values of the SeNPs both before and after their interaction with EPS. Notably, SeNPs–EPS exhibited a higher Zeta potential value (−29.34 ± 6.3 mv) compared to SeNPs (−19.59 ± 8.2 mv). This Z-average is attributed to the enhanced electrostatic repulsion between nanoparticles. The elevated Zeta potential value is indicative of greater stability [55]. Typically, the zeta potential represents the charges on the surface of NPs (negative or positive) and their magnitude. It is often varying, according to the kind of ligands used during the preparation. Usually, nanoparticles with near-neutral zeta potential or mildly charged surfaces tend to aggregate faster, which implies that the stronger the charge, the better is the colloidal stability of the particles [56]. According to the DLS pattern, SeNPs and SeNPs–EPS ranged in size from 200 to 300 nm and 200 to 400 nm, respectively, and 52% of the sample has an average size between 209 and 328 nm (Figure 4c,d).

**Figure 5:**
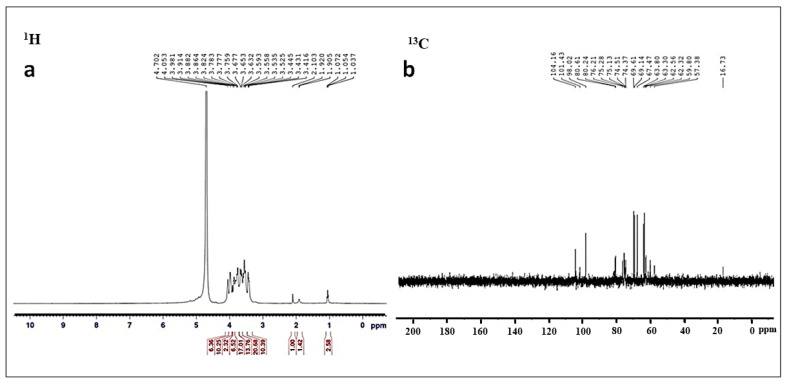
NMR characterization strain MKUST-01 EPS: (**a**) ^1^H NMR spectrum and (**b**) ^13^C NMR spectrum.

The results of ^1^H and ^13^C-NMR spectra of EPS revealed the complex structure and heterogeneous nature of EPS (Figure 5a,b). A prominent chemical shift in ^1^H NMR (3.83, 3.72, and 3.62 ppm) suggested that the polysaccharide of the isolate MKUST-01 is a hetero polysaccharide, and this observation aligns with a prior report by Sathishkumar et al. [57] that extracted the EPS from marine sponge-associated *B. subtilis* MKU SERB2.

**Figure 6:**
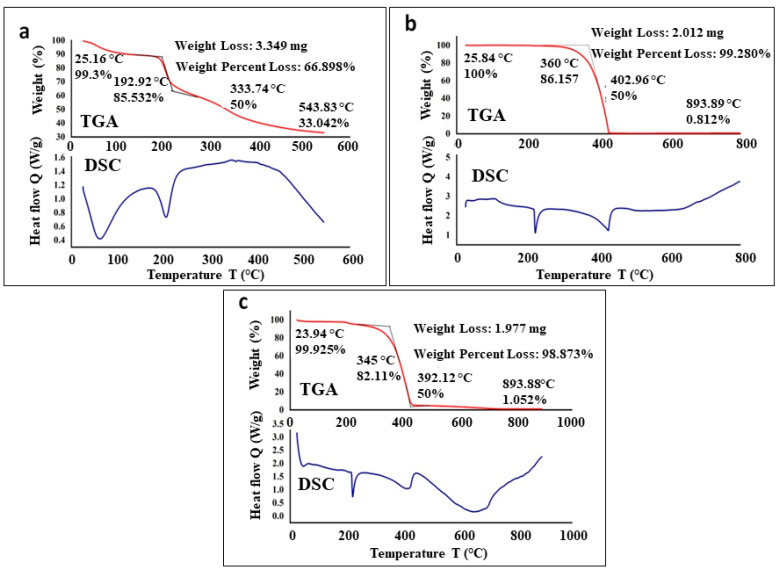
TGA and DSC analysis of bacterial (**a**) EPS, (**b**) SeNPs, and (**c**) SeNPs–EPS conjugate.

The stability of EPS, SeNPs, and SeNPs–EPS was assessed using thermogravimetry, a valuable technique for monitoring a materials’ weight loss as temperature varies. The process of thermal degradation of EPS involves heat emission and absorption, which is concomitant with alterations in the polymer’s structure and melting of crystalline polymer segments [58]. A degradation temperature of 192.9 °C was determined for EPS (cf. Figure 6a, DSC). An initial weight loss (~10%) between 50 and 105 °C was attributed to moisture and alcohol content trapped in the EPS. The higher levels of alcohol and moisture can be attributed to the abundance of carboxyl groups present in EPS. With increasing temperature, a dramatic weight loss (about 40%) was observed between ~180 and 210 °C, which is due to the actual degradation of EPS. Furthermore, the complete weight loss of EPS occurs after 500 °C. The DSC and TGA analysis of pure SeNPs were recorded from 25° to 800 °C. In the TGA thermogram, only a single step, highly steep peak is observed at ~402 °C, indicating the complete weight loss (almost 100%) of SeNPs (cf. Figure 6b, TGA). The sample did not show any moisture content as reflected by the absence of any weight loss below 100 °C. On the other hand, the DSC thermogram of the synthesized SeNPs exhibited a small exothermic transition peak at ~90 °C, along with a sharp endothermic melting peak at ~230 °C (cf. Figure 6b, DSC). With further increasing temperature, around complete weight loss at a temperature in between 360 and 450 °C. In comparison with pure EPS and pure SeNPs, the TGA and DSC thermogram of SeNPs–EPS demonstrated a mixed behavior. For instance, unlike in pure SeNPs, the TGA of SeNPs–EPS shows slight weight loss below 50 and 105 °C (Figure 6c, TGA), which is possibly due to the residual EPS contents. Furthermore, a sharp endothermic melting peak at ~230 °C belonging to the SeNPs is slightly shifted to a lower temperature and exists at ~210 °C due to the existence of EPS on the surface of NPs (cf. Figure 6c, DSC). Thereafter, the complete degradation of SeNPs–EPS occurred between 360 and 450 °C.

### 3.3. Biological Activities

#### DPPH and Reducing Power Assay

**Figure 7:**
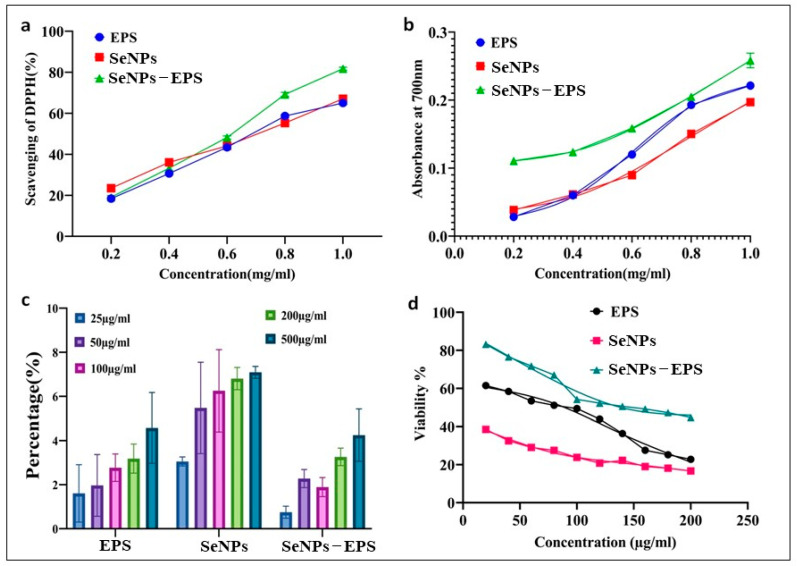
Biological activities of bacterial EPS, SeNPs, and SeNPs–EPS conjugates: (**a**) scavenging activity of DPPH, (**b**) total antioxidant assay, (**c**) hemolytic activity, and (**d**) MTT cell viability assay.

The antioxidant potentials of EPS, SeNPs, and SeNPs–EPS conjugates were measured using DPPH and total reducing power assay. The scavenging capacity of the EPS, SeNP, and SeNPs–EPS increased in the concentration range between 0 and 1.0 mg/mL as given in Figure 7a. Maximum activity of 82 ± 0.12% was observed with the concentration 1 mg/mL of SeNPs–EPS, in contrast with other samples. Increasing the concentration from 1 to 1.5 mg/mL could not significantly enhance the scavenging effect. Likewise, EPS from Leuconostoc mesenteroides WiKim32 exerts the scavenging activity on DPPH radicals and ferric ions reducing antioxidant power. Chen et al. [59] reported SeNPs stabilized with chitosan (50 nm size) exhibited significant DPPH and ABTS scavenging abilities. Additionally, Peng et al. [60] found that SeNPs ranging size from 5–200 nm were capable of directly scavenging free radicals in vitro, and this activity was shown to depend on the size of the SeNPs, understanding the importance of NPs size in their biological activity. The total reducing power of SeNPs and SeO_2_ demonstrated a dose-dependent trend within the concentration range of 0–1 mg/mL (Figure 7b). Across all concentrations, it is worth noting that the reducing power of EPS and SeNPs–EPS were notably lower than that of SeNPs (*p* < 0.05). However, when the concentration exceeded 1 mg/mL, then the reducing power of both EPS and SeNPs–EPS became significantly higher than that of SeNPs (*p* < 0.05). This increase in reducing power can be attributed to the solubility of EPS [54,61].

Figure 7c represents the hemolytic activity of EPS, SeNPs, and SeNPs–EPS. The result showed that tested samples have no hemolytic properties. The results of human embryonic kidney cells (HEK-293) viability were shown in Figure 7d. IC50 concentrations of EPS and SeNPs–EPS were 138.492 mg/mL and 89.028 mg/mL, respectively, and significantly (*p* < 0.05) less toxic to SeNPs, whereas, SeNPs showed 50% cell inhibition at 6.729 mg/mL. These results are in contrast to the findings of Forootanfar et al. [62], who reported that SeNPs produced by *Bacillus* sp. MSh-1 were less toxic to the MCF-7 cell line compared to SeO_2_ ions. However, their research found that the EPS-conjugated SeNPs were less hazardous than SeNPs on the normal HEK cell line. Prior in vivo research [63] has also reported that SeNPs are less hazardous than selenite ions. The determined IC50 for EPS-conjugated SeNPs and SeNPs were compared, and it was shown that EPS-conjugated SeNPs were around 20 times less harmful for the HEK cell line. The EPS does not show any substantial cytotoxicity even at high concentrations (10 mg/mL), which reveals the SeNPs conjugated with EPS has reduced toxic effects on the cells.

### 3.4. In Vivo Toxicity Study

#### Gnotobiotic Brine Shrimp

**Figure 8:**
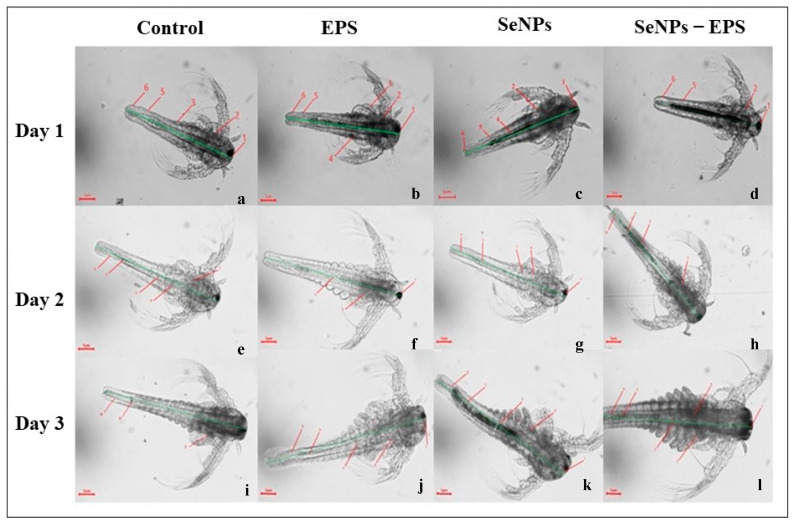
Gnotobiotic Artemia assay (microscopic images of *A. nauplii* larval development under 40× magnification). Illustrative light microscopic metaphors showing developmental stages of *A. nauplii* larvae from day 1 in response to probiotic treatments and without treatment (**a**–**d**), day 2 (**e**–**h**), and day 3 (**i**–**l**). Control, EPS, SeNPs, and SeNPs–EPS. (Note: Various developmental stages of Artemia nauplii larvae were represented in numerical: 1—foregut endothelial cells, 2—brush border membrane, 3—intestine, 4—midgut endothelial cells, 5—midgut-hindgut transition, 6—hindgut endothelial cells.

Gnotobiotically cultured brine shrimp *Artemia nauplii* were utilized for in vivo toxicity assessment. Notably, there was a significant increase in the survival rate of *Artemia nauplii* treated with EPS (76 ± 5%; *p* ≤ 0.0004) and SeNPs–EPS (77 ± 5%; *p* ≤ 0.007), in contrast to the untreated group, where the survival rate was only 52 ± 1.2% on day 3 (Figure 8). Conversely, a significant reduction in the survival rate was observed in the SeNPs-treated group (58 ± 0%; *p* ≤ 0.002) compared to the untreated group (Table 2). Additionally, there was an enhancement in the individual length of the treated groups SeNPs–EPS and EPS compared to SeNPs (Figure 8). The brine shrimps exhibited rapid growth with distinct segments, including feeding appendages and a mid-hindgut transition observed in the EPS-treated group on day 1. These results align with a previous study involving the probiotic bacteria *Exiguobacterium acetylicum* S01 [64]. Notably, the leaf-like appendages displayed prominent growth, contributing to the evident development of the brine shrimp A. nauplii in response to SeNPs–EPS, while the untreated *A. nauplii* group exhibited just the initial appearance of somites (Figure 8).

## 4. Conclusions

The current study highlights the significant potential of EPS and SeNPs in the realm of biomedicine and nanotechnology. The investigation underscores the multifaceted biological attributes of EPS, encompassing antibacterial, antioxidant, and anticancer effects. Moreover, the utilization of polysaccharide-coated nanoparticles, which exhibit exceptional bioactivity, showcases their versatility in therapeutic and diagnostic applications. The deliberate synthesis and conjugation of SeNPs with EPS derived from *Bacillus* sp. MKUST-01 has been meticulously characterized through various analytical techniques. UV–Vis spectral exhibited unique peaks, and XRD as well as FTIR spectroscopy offered valuable information regarding the crystalline properties of selenium and the identification of functional groups within the EPS layer. The morphological assessment of SeNPs depicted elongated rod-like structures with promising dimensions, which enhances their potential for various applications.

An intriguing finding is the absence of hemolytic activity exhibited by SeNPs–EPS conjugates, a contrast to SeNPs acting alone. Additionally, the SeNPs–EPS conjugates showcased an impressive 20% enhancement in antioxidant capacity, outperforming both SeNPs and EPS. The promising outcomes extend further into the domain of gnotobiotic Artemia nauplii, where the supplemented EPS and SeNPs–EPS conjugates contributed to improved growth and heightened survival, a significant observation compared to SeNPs-fed Artemia nauplii subjected to a microalgal diet. The amalgamation of biologically derived EPS and nano-engineered SeNPs emerges as a potential avenue for advancing therapeutic interventions. These findings open up avenues for further exploration, including understanding the mechanistic underpinnings of the observed phenomena and potential applications in medical and environmental contexts. The convergence of biotechnology, nanotechnology, and biomedical sciences presented here contributes to the evolving landscape of innovative solutions that bridge cutting-edge research with tangible outcomes.

## Figures and Tables

**Table 1:** EPS production of the selected bacterial isolates.

Isolates (Strains)	Biomass (gm/L)	Crude Preparation			Partially Purified	
		EPS (g/L)	Carbohydrate (mg/g)	Protein (mg/g)	EPS (g/L)	Carbohydrate (%)
MKUST03	1.73 ± 0.10	3.03 ± 0.34	60.66 ± 1.52	58.00 ± 0.98	0.891 ± 0.52	89.00 ± 1.14
MKUST04	5.9 ± 0.39	2.78 ± 0.20	43.00 ± 2.64	23.00 ± 0.22	0.826 ± 0.11	71.00 ± 0.96
MKUST15	7.01 ± 0.34	1.41 ± 0.27	86.33 ± 2.51	72.00 ± 0.15	0.502 ± 0.01	82.00 ± 0.22
MKUST19	1.63 ± 0.07	1.38 ± 0.17	71.33 ± 2.30	64.00 ± 0.78	0.317 ± 0.058	87.00 ± 0.05
MKUST25	0.51 ± 0.24	1.52 ± 0.37	59.23 ± 8.02	72.00 ± 0.241	0.243 ± 0.021	82.00 ± 1.06
MKUST29	0.93 ± 0.045	1.35 ± 0.16	29.45 ± 3.56	76.00 ± 1.02	0.14 ± 0.06	72.00 ± 0.09
MKUST30	1.24 ± 0.10	1.42 ± 0.39	52.34 ± 2.08	56.00 ± 1.18	0.252 ± 0.12	70.00 ± 1.0

**Table 2:** Gnotobiotic Artemia assay.

Sampling Day	Survival Rates (%) *				Individual Length (µm)			
	Control	EPS	SeNPs	SeNPs–EPS	Control	EPS	SeNPs	SeNPs–EPS
1	94 ± 0.4	96 ± 1.2	87 ± 1.0	95 ± 1	85 ± 0.4	82.5 ± 6.6	57.5 ± 3.6	74.2 ± 1.02
2	75 ± 0.9	83 ± 0.8	78 ± 0.2	94 ± 0	86 ± 0.26	92.5 ± 0.2	85. ± 9.2	95. ± 2.6
3	52 ± 1.2	76 ± 1	58 ± 0	77 ± 5	92 ± 0.71	97.5 ± 1.6	92.5 ± 1.9	100 ± 4.2

* Survival rate of and the length of the *Artemia nauplii*. Values are mean of three individual experiments with ±SD.

## Data Availability

Data contained within the article and supplementary file.

## Supplementary Materials

The following supporting information can be downloaded at: https://www.mdpi.com/article/10.3390/biomedicines11092520/s1, Figure S1: String test and Congo red agar plates showed EPS-producing bacterial isolates.
