# Supplementary material for: Synthesis and Structural Characterization of Selenium Nanoparticles–Bacillus sp. MKUST-01 Exopolysaccharide (SeNPs–EPS) Conjugate for Biomedical Applications
Source: Biomedicines. 2023 Sep 12;11(9):2520. doi: 10.3390/biomedicines11092520 (PMC10525696; doi:10.3390/biomedicines11092520)
Supplement: Supplementary file 1 [file biomedicines-11-02520-s001.zip › biomedicines-2563062-supplementary.pdf]

biomedicines

MDPI

Supplementary File

Synthesis and Structural Characterization of Selenium Nanoparticles – *Bacillus* sp. MKUST-01 Exopolysaccharide (SeNPs-EPS) Conjugate for Biomedical Applications

Thirumalaivasan Ramachandran [1], Devaprakash Manoharan [1], Sivakumar Natesan [1,*], Shyam Kumar Rajaram [2], Ponmurugan Karuppiah [3], Mohammed Rafi Shaik [4,*], Mujeeb Khan [4] and Baji Shaik [5]

1. Department of Molecular Microbiology, School of Biotechnology, Madurai Kamaraj University, Madurai 625021, Tamilnadu, India; thivasan7498@gmail.com (T.R.); devprakashm3@gmail.com (D.M.)
2. Department of Biotechnology, Kamaraj College of Engineering and Technology, Virudhunagar 625701, Tamil Nadu, India; kingshyam2003@gmail.com
3. Department of Botany and Microbiology, College of Science, King Saud University, Riyadh 11451, Saudi Arabia; pkaruppiah@ksu.edu.sa
4. Department of Chemistry, College of Science, King Saud University, P.O. Box 2455, Riyadh 11451, Saudi Arabia; kmujeeb@ksu.edu.sa
5. School of Chemical Engineering, Yeungnam University, Gyeongsan 38541, Republic of Korea; shaikbaji@yu.ac.kr

* Correspondence: siva.biotech@mkuniversity.ac.in (S.N.); mrshaik@ksu.edu.sa (M.R.S.)

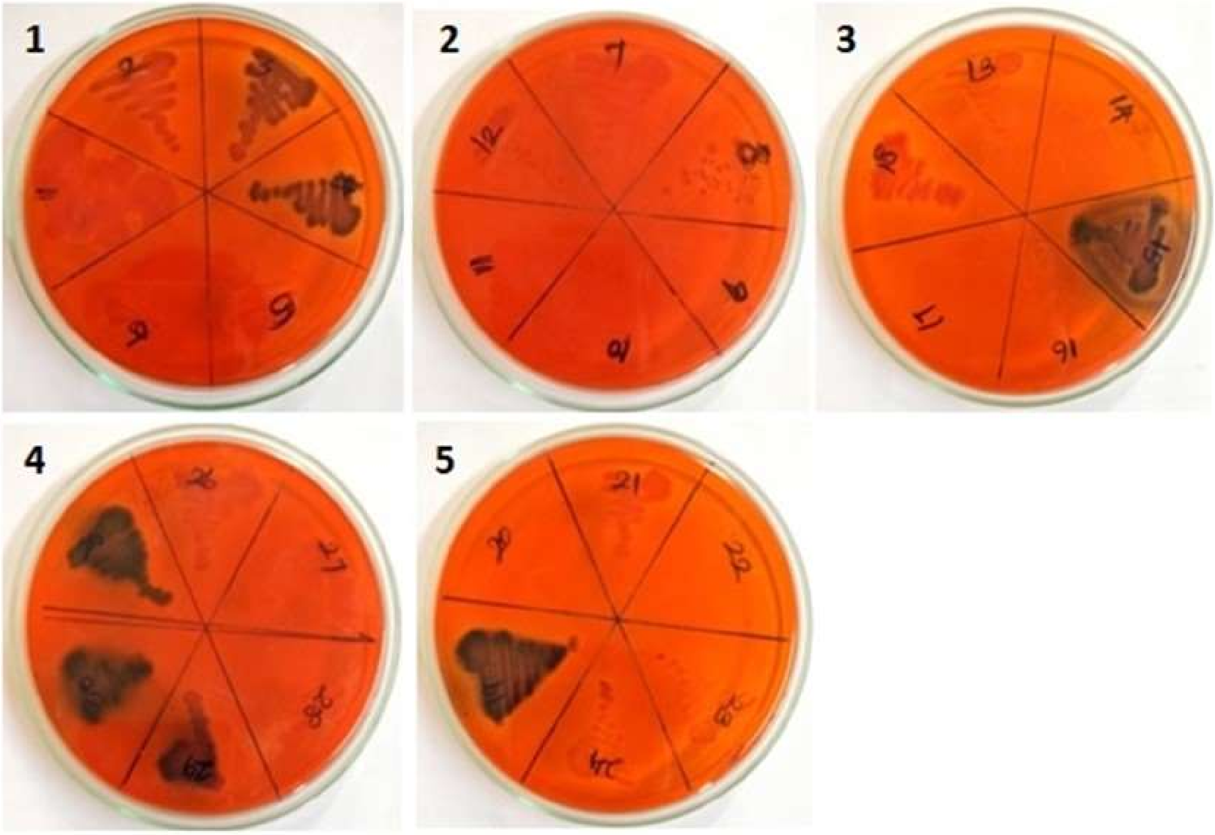

Figure S1. String test and Congo red agar plates showed EPS producing bacterial isolates.
